# Biomechanical and movement-quality predictors of lower-limb load-related injury in collegiate athletes: a prospective cohort study using functional movement screening, wearable inertial sensing, and explainable machine learning

**DOI:** 10.3389/fphys.2026.1920109

**Published:** 2026-09-30

**Authors:** Xuqingfeng Xu, Jihe Zhang, Jianlu Liao

**Affiliations:** Department of Aviation Sports, Aviation University of Air Force, Changchun, China

**Keywords:** biomechanical asymmetry, collegiate athletes, explainable machine learning, functional movement screen, inertial measurement unit, injury prediction, prospective cohort, SHAP

## Abstract

Lower-limb load-related injuries account for a disproportionate share of time loss in collegiate sport, yet the biomechanical and movement-quality factors distinguishing injured from uninjured athletes remain incompletely characterized. This prospective cohort study followed 161 collegiate athletes for one competitive season, integrating Functional Movement Screen task-level scores with wearable inertial measurement unit-derived features — including peak tibial acceleration asymmetry, surrogate vertical loading rate, and pelvis–trunk stability indices — within an explainable machine learning framework. Four classifiers were compared across FMS-only, wearable-only, and fused configurations under nested five-fold cross-validation with embedded LASSO feature selection. Thirty-seven load-related time-loss injuries were recorded (23.0% incidence). The fused XGBoost model achieved an out-of-fold AUC of 0.70 (95% CI: 0.61–0.79), outperforming FMS-only (AUC 0.62) and wearable-only (AUC 0.64) configurations by statistically significant margins. Peak tibial acceleration asymmetry and surrogate loading rate on the non-dominant limb were the strongest biomechanical predictors; in-line lunge and active straight-leg raise task scores contributed most among FMS inputs. Biomechanical features added an independent AUC increment of 0.07 beyond previous-injury history. SHAP analysis identified an interaction between tibial impact asymmetry and in-line lunge score that met the pre-specified directional stability criterion. These findings establish a physiologically interpretable template for biomechanical phenotyping of injury risk, supporting the combined use of wearable sensing and task-level movement screening in collegiate athletic populations.

## 1 Introduction

Lower-limb injuries remain among the most common and costly problems in collegiate sport, accounting for a disproportionate share of time lost from training and competition across more than a decade of surveillance data (Hootman et al., 2007). Yet the way these injuries are framed matters. Many of the most prevalent presentations in physically active populations — tibial stress injuries, tendinopathies of the patellar and Achilles tendons, and recurrent soft-tissue strains — are not isolated accidents but the visible endpoint of a slower physiological process, in which repeated mechanical loading outpaces the capacity of bone and connective tissue to adapt (Hoenig et al., 2022). Bone responds to habitual loading through continuous remodeling, but the reparative phase lags behind microdamage accumulation, so injury becomes likely when recovery windows are too short for adaptation to catch up (Warden et al., 2021). Tendon behaves in a comparable way: collagen synthesis and structural reorganization follow mechanical stimulation only after a measurable delay, and the magnitude of that response differs between individuals (Magnusson et al., 2008; Kjaer et al., 2009). Understood in these terms, load-related injury is best read as a mismatch between cumulative mechanical exposure and the neuromuscular and tissue-level capacity to tolerate it. The same logic holds at the level of whole-athlete monitoring, where sharp increases in workload relative to an athlete’s chronic baseline track with elevated injury risk (Gabbett, 2016), and where the balance between acute and chronic loading appears to mark a window within which tissue is neither under-prepared nor overwhelmed (Maupin et al., 2020). Risk, on this account, is less a fixed attribute of an athlete than a function of exposure weighed against the ability to control and absorb it.

If injury reflects this exposure–capacity balance, the tools used to anticipate it ought to capture both sides of the equation — and it is here that conventional movement screening shows its limits. The Functional Movement Screen (FMS) was devised to flag deficits in fundamental movement patterns, yet its capacity to forecast injury has proven inconsistent. A composite score at or below the conventional threshold of 14 has been linked to roughly a 2.7-fold increase in injury odds in pooled analysis (Bonazza et al., 2017), whereas other syntheses report only a small association, insufficient to justify the screen as a stand-alone predictive instrument (Moran et al., 2017). The discriminative ceiling is modest in absolute terms, with meta-analytic estimates of the area under the curve clustering near 0.59 and sensitivity frequently below 0.30 even where specificity is high (Dorrel et al., 2015; Moore et al., 2019). Much of this disagreement traces to heterogeneity in cut-points, injury definitions, and populations, which leaves the pooled evidence statistically unstable (Trinidad-Fernández et al., 2019). Part of the difficulty, however, may be structural rather than merely empirical. Collapsing seven distinct movement tasks into a single summed score discards information about which specific patterns are impaired, and recent appraisals argue that such composite indices obscure the individual movement-quality signals that carry physiological meaning (Eckart et al., 2025). Seen through the load–capacity lens, then, the problem is not only that the FMS predicts weakly; it is that the summed score compresses precisely the neuromuscular-control information one would most want to keep.

Wearable inertial measurement units (IMUs) offer a complementary signal, one grounded in what the body actually does under load rather than what it can do on demand. Tibial-mounted accelerometers quantify impact magnitude, loading rate, and inter-limb asymmetry during real movement, supplying a peripheral proxy for the mechanical exposure that drives the adaptive process outlined above (Sheerin et al., 2019). The link between such surface-measured signals and the internal stresses borne by bone is nonetheless far from direct, and treating tibial acceleration as a literal index of bone load overstates what the sensor can resolve (Xiang et al., 2024). The signal is therefore better regarded as a surrogate for impact loading — informative, but in need of physiological anchoring. A further obstacle is that the two streams — capacity-oriented information from movement screening, exposure-oriented information from wearable sensing — are usually analyzed in isolation, even though they are conceptually two halves of one balance. And because injury emerges from non-linear interactions among many partial contributors rather than from any single dominant cause, linear, single-source models are poorly suited to integrating them.

Computational methods are well placed to close this gap, since machine learning can accommodate non-linear, multi-source relationships and, when paired with *post-hoc* interpretation, can convert predictive structure into mechanistic insight. Systematic appraisals of machine learning for injury prediction nonetheless caution that reported performance is often overstated, and that data leakage — allowing observations from the same athlete to fall in both training and test sets, for example — inflates apparent accuracy (Van Eetvelde et al., 2021). Work on artificial intelligence in team-sport injury risk makes a parallel point: what the field most needs is methodological standardization, not algorithmic novelty alone (Claudino et al., 2019). A recent scoping review charting this literature found reported AUCs spanning 0.57 to 0.95, yet noted that only about 18% of studies adopted any form of model explainability and merely 13% used Shapley-based attribution (Leckey et al., 2025) — a notable shortfall, given that interpretability is what would allow such models to speak to physiology rather than to prediction alone. Framing injury as a complex, dynamically interacting system strengthens the case for exactly this pairing: non-linear models able to represent interactions, coupled with attribution methods that expose them (Bittencourt et al., 2016). Shapley additive explanations provide a principled basis for that attribution, decomposing a model’s output into the contribution of each feature (Lundberg and Lee, 2017). Building on this, the present study sets out not to maximize predictive accuracy for its own sake but to identify quantifiable biomechanical and movement-quality factors that carry the signal of lower-limb load-related injury, and to map those factors back onto exercise-physiological mechanisms. We pursue three aims: first, to identify candidate predictors and interpret their physiological meaning; second, to compare the discriminative performance of FMS-only, wearable-only, and fused data configurations, and to test whether fusion adds information beyond previous-injury history and what such an increment would mean physiologically; and third, to explore, in a deliberately tentative manner, the cross-source interactions between movement-screening and wearable-derived features. To our knowledge, no prospective collegiate cohort study has yet integrated FMS-derived movement quality with wearable impact-loading biomechanics within an ablation- and SHAP-evaluated framework that explicitly maps predictors onto the physiological mechanisms of load-related injury.

## 2 Materials and methods

### 2.1 Study design, analysis plan, and participants

This study was designed as a single-season prospective cohort and is reported following the STROBE statement for observational research (von Elm et al., 2007) and, for the predictive-modeling components, the TRIPOD+AI checklist for regression and machine-learning prediction studies (Collins et al., 2015; Collins et al., 2024). The analysis plan — covering the outcome definition, candidate feature set, modeling pipeline, and primary comparisons — was fixed before any outcome data were examined, guarding against *post-hoc* hypothesis generation. A total of 168 collegiate athletes (94 men, 74 women; age 20.4 ± 1.6 years) were recruited from university teams competing in sports with substantial running and cutting demands, including soccer, basketball, and track and field. Eligibility required active training and competition status at enrolment; athletes carrying an unresolved lower-limb injury at baseline or unable to complete the preseason assessment were excluded. Preseason testing took place during the two weeks before the competitive season, after which participants were followed for approximately 30 weeks. Athletes who withdrew for non-injury reasons were censored at the time of withdrawal and excluded from the primary analysis.

### 2.2 Functional movement assessment

Movement quality was characterized using the seven-task Functional Movement Screen, with each task scored on a 0–3 ordinal scale (composite range 0–21). Rather than collapsing performance into a summed total, all seven task scores were retained as separate model inputs, since doing so preserves the pattern-specific information that a single composite obscures. Two certified raters conducted screening under a standardized protocol; inter-rater reliability was estimated in a random subsample, yielding agreement consistent with the pooled intraclass correlation coefficient of 0.81 reported in prior meta-analytic work on this instrument (Bonazza et al., 2017).

### 2.3 Wearable inertial monitoring and biomechanical feature derivation

Each athlete was fitted with three IMUs: one positioned at the sacrum (L5–S1) and one over the distal anteromedial aspect of each tibia, approximately 1 cm proximal to the medial malleolus, with the sensor axis aligned to the tibial shaft and secured to limit soft-tissue artefact. Tibial sensors sampled at 1000 Hz to capture the brief transients of impact loading; the sacral sensor operated at 200 Hz, sufficient for the lower-frequency kinematics of pelvis–trunk control. Sensor-to-segment alignment was established via combined static and functional calibration before data collection, consistent with established field-based inertial gait analysis practice (Berner et al., 2020).

Three families of biomechanical features were derived from the raw signals. Impact-loading variables included axial peak tibial acceleration and surrogate vertical loading rate. Inter-limb asymmetry was quantified using a symmetry index defined as:


$$ASI=\frac{\left|X_{dom}-X_{non}\right|}{\frac{1}{2}\left(X_{dom}+X_{non}\right)}\times 100\%$$

where *X_dom_* and *X_non_* denote the value of a given metric for the dominant and non-dominant limb, respectively. Dominant limb was defined as the self-reported preferred kicking or jumping leg, consistent with standard practice in asymmetry research. Pelvis–trunk stability was indexed from sacral angular-velocity and orientation measures. Throughout, tibial acceleration is treated as a peripheral surrogate for impact loading rather than a direct measure of internal bone stress, since the correspondence between surface-measured acceleration and the forces actually borne by cortical bone remains partial (Sheerin et al., 2019; Xiang et al., 2024) — a boundary made explicit here so that the physiological interpretation offered later stays within what three body-worn sensors can reasonably resolve.

### 2.4 Injury surveillance and outcome definition

The primary outcome was the first lower-limb load-related time-loss injury incurred during the follow-up season. A time-loss event was defined as absence of at least one day from training or competition combined with inability to complete at least one subsequent session, consistent with recommendations favoring unambiguous, meaningful loss-of-participation criteria (Moore et al., 2019). Eligible injury types were those plausibly arising through the load–capacity pathway under investigation: tibial stress injuries, foot and ankle overuse conditions, patellar and Achilles tendinopathies, and hamstring or calf strains. Acute contact trauma was excluded, as its mechanism falls outside what the present measurement framework can address. Injuries were recorded and adjudicated by team medical and strength-and-conditioning staff, with absence duration cross-checked against attendance logs.

### 2.5 Feature boundary, covariates, and selection

Biomechanical predictors — comprising the IMU-derived loading and kinematic variables together with the seven FMS task scores — were distinguished from contextual covariates (age, sex, BMI, and previous-injury history). This distinction reflects the central analytical question: whether biomechanical features contribute information beyond the well-established predictive weight of prior injury, an increment that is informative regardless of its direction. After preprocessing, the candidate matrix contained 168 observations across 23 features. Given the limited event count anticipated for a single competitive season, the number of retained predictors was constrained; feature selection was performed using LASSO (Tibshirani, 1996) and embedded entirely within the inner loop of cross-validation, so that held-out fold data never informed which features were selected — a precaution that matters because selection performed on the full sample introduces optimism that inflates apparent performance (Varma and Simon, 2006). The events-per-variable ratio was reported transparently at each stage rather than assumed to meet a fixed threshold (Peduzzi et al., 1996). The proportion of inner-loop folds in which each candidate feature was retained by LASSO was recorded and is reported in **Table 1** to allow readers to assess selection stability.

**Table 1:** Top-ranked predictors by mean absolute SHAP value from the fused XGBoost model.

Feature	Source	Sel. freq. (%)	Mean |SHAP|	Direction	Physiological note
Previous-injury history	Covariate	100	0.31	↑	Accumulated tissue vulnerability; structural and motor residuals from prior damage
ASI-PTA	IMU	92	0.24	↑	Inter-limb asymmetry in peak tibial acceleration; surrogate for unequal load distribution
Surrogate loading rate (non-dominant)	IMU	84	0.19	↑	Monotonic risk increase above 1.8 g·ms^−^¹; consistent with load–adaptation mismatch
In-line lunge (ILL) score	FMS	80	0.17	↑ (score = 1)	Frontal-plane stability and hip/ankle mobility deficit under split-stance demand
Active straight-leg raise (ASLR) score	FMS	72	0.14	↑ (score = 1)	Posterior chain extensibility asymmetry; sensitive to unilateral flexibility limitation
Sacral angular-velocity range	IMU	64	0.12	↑	Index of pelvis–trunk stability; elevated range reflects dynamic control deficit
Deep squat score	FMS	48	0.09	↑ (score = 1)	Bilateral mobility and stability; modest independent contribution beyond ILL/ASLR
Age	Covariate	40	0.06	↑	Modest positive association; likely reflects cumulative loading history
Sex	Covariate	32	0.05	Mixed	Directionally inconsistent across folds; retained as feature but not independently interpreted
BMI (kg/m²)	Covariate	24	0.04	↑	Weak positive association; not retained in all folds after LASSO selection

Features ranked by mean absolute SHAP value from the fused XGBoost model. Direction indicates whether higher feature values were associated with increased (↑) or decreased (↓) injury probability in SHAP dependence plots. ASI-PTA, peak tibial acceleration asymmetry index; ILL, in-line lunge; ASLR, active straight-leg raise; IMU, inertial measurement unit; FMS, Functional Movement Screen. Selection frequency (Sel. freq.) denotes the percentage of inner-loop LASSO folds (n = 25) in which each feature was retained with a non-zero coefficient. Features retained in ≥ 80% of folds are considered robust; those below 50% should be interpreted with caution.

### 2.6 Predictive modeling

Four classifiers were compared on equal terms: regularized logistic regression as an interpretable benchmark, random forest, XGBoost, and a support vector machine. No prior assumption was made that ensemble methods would outperform the linear model, as the evidence for such superiority in injury prediction is inconsistent. Performance was estimated via nested five-fold cross-validation, with hyperparameter tuning confined to the inner loop and evaluation to the outer loop, an arrangement that prevents the selection process from inflating test-set estimates (Varma and Simon, 2006). Class imbalance was addressed by applying SMOTE (Chawla et al., 2002) within each training fold only, ensuring synthetic observations never appeared in evaluation data; models trained without resampling were retained as a sensitivity check. Sex was included both as a feature in the pooled model and examined in stratified analyses, given evidence that sex-specific models can offer meaningful performance gains (Bogaert et al., 2022).

### 2.7 Ablation experiments

Three data-source configurations were constructed and compared: FMS-only, wearable-only, and a fused model combining both. Pairwise differences in discriminative performance were tested using the DeLong method for correlated areas under the curve (DeLong et al., 1988). To ensure that any fusion advantage reflected genuine informational complementarity rather than increased feature dimensionality alone, the number of retained features was reported for each configuration alongside its performance metrics.

### 2.8 Explainability and interaction analysis

Discriminative performance was evaluated across all four classifiers, but model interpretation was restricted to the best-performing tree-based learner. This choice is motivated by the requirement of Shapley-based attribution methods — and interaction decomposition in particular — for a model structure capable of capturing non-additive relationships. Global feature importance and individual dependence plots were derived using SHAP (Lundberg and Lee, 2017), which apportions each prediction across features according to their average marginal contribution across all possible feature subsets. Cross-source interaction effects were treated as exploratory throughout: directional stability was assessed via permutation testing and resampled subsets, and interactions that did not meet the pre-specified stability criterion are reported without substantive interpretation. The model used for explanation was not required to be the same as the best-discriminating model; conflating the two would overstate the inferential weight of the interaction analysis. A tree-based learner was chosen for this role because SHAP interaction values are structurally zero in an additive model unless interaction terms are pre-specified, making a non-additive architecture a prerequisite for the exploratory interaction analysis. The regularized logistic regression coefficients and odds ratios are reported in **Supplementary Table 1** as a model-independence check. A schematic of the SHAP decomposition workflow is provided in **Supplementary Figure 2**.

### 2.9 Internal validation

The primary validation approach relied on out-of-fold predictions aggregated across the nested cross-validation. A chronological split — training on early-season data and testing on later events — was planned as a supplementary sensitivity check; whether it yields sufficient events to be informative is reported transparently in the Results. No external validation cohort was available, a limitation acknowledged in the Discussion.

### 2.10 Evaluation metrics and statistical analysis

Discrimination was summarized by the area under the ROC curve as the primary metric, with the area under the precision–recall curve (PR-AUC), sensitivity, specificity, and F_1_ score reported as complements. Because synthetic oversampling distorts predicted-probability distributions, calibration was assessed on the original, non-resampled class distribution and quantified using the Brier score (Steyerberg et al., 2010). Per-fold positive-event counts and confidence intervals were reported throughout to keep the statistical limits imposed by event count visible. Between-configuration AUC differences were tested with the DeLong method (DeLong et al., 1988), with two-sided α = 0.05. No correction for multiple comparisons was applied across the three pairwise DeLong tests, given their pre-specified nature and the exploratory context of the ablation analysis; results are interpreted accordingly.

This study was approved by the Ethics Committee of Aviation University of Air Force (AUAF-EC-2024-403; 08 May 2024). The study was conducted in accordance with the Declaration of Helsinki (1975, revised 2013) and relevant national and institutional guidelines concerning human participant research. All participants provided written informed consent prior to enrolment.

## 3 Results

### 3.1 Participant characteristics and injury incidence

Of the 168 athletes enrolled at preseason, 161 completed the full competitive season and were retained in the primary analysis. Seven withdrew for non-injury reasons (academic withdrawal, n = 4; voluntary discontinuation, n = 3) and were censored at the time of withdrawal. The analytical cohort comprised 90 men and 71 women with a mean age of 20.4 ± 1.6 years, mean BMI of 22.8 ± 2.4 kg/m², and a mean FMS composite score of 15.1 ± 2.1 (range: 9–20). Inter-rater agreement for FMS scoring, estimated in a random subsample of 28 athletes, yielded an ICC of 0.83 (95% CI: 0.76–0.89). Forty athletes (24.8%) reported a lower-limb injury in the 12 months preceding enrolment.

Over the 30-week season, 37 first lower-limb load-related time-loss injuries were recorded across 37 athletes, corresponding to a seasonal incidence of 23.0% (95% CI: 16.6–30.3%). By subtype, the distribution was: patellar and Achilles tendinopathies (n = 11, 29.7%), tibial stress injuries (n = 9, 24.3%), hamstring and calf strains (n = 9, 24.3%), and foot and ankle overuse conditions (n = 8, 21.6%). A further 14 acute contact injuries were excluded per protocol. Median time to injury onset was 11.4 weeks (IQR: 7.2–18.6 weeks). Previous injury was more prevalent among injured athletes (17 of 37, 45.9%) than among those who remained injury-free (23 of 124, 18.5%), a difference that was statistically significant (*χ*^2^(1) = 11.43, *p* < 0.001). Baseline characteristics stratified by injury status are presented in **Table 2**.

**Table 2:** Baseline characteristics of the analytical cohort (n = 161) stratified by injury status.

Variable	Full cohort (n = 161)	Injured (n = 37)	Non-injured (n = 124)
Demographics			
Age (years)	20.4 ± 1.6	20.6 ± 1.7	20.3 ± 1.6
Sex, male, n (%)	90 (55.9)	19 (51.4)	71 (57.3)
BMI (kg/m²)	22.8 ± 2.4	23.1 ± 2.5	22.7 ± 2.3
Preseason screening			
FMS composite score	15.1 ± 2.1	14.6 ± 2.3	15.3 ± 2.0
FMS composite ≤ 14, n (%)	48 (29.8)	15 (40.5)	33 (26.6)
Previous injury history, n (%)	40 (24.8)	17 (45.9)	23 (18.5)
Injury characteristics (injured group only)			
Median time to injury (weeks)	—	11.4 (7.2–18.6)	—
Tibial stress injuries, n (%)	—	9 (24.3)	—
Foot/ankle overuse, n (%)	—	8 (21.6)	—
Patellar/Achilles tendinopathy, n (%)	—	11 (29.7)	—
Hamstring/calf strain, n (%)	—	9 (24.3)	—

Values are mean ± SD unless otherwise stated. Time to injury shown as median (IQR). Previous injury: lower-limb time-loss injury in the 12 months preceding enrolment. BMI, body-mass index; FMS, Functional Movement Screen; IQR, interquartile range.

With 37 outcome events across 23 candidate features, the pre-selection events-per-variable ratio was 1.6. Following LASSO-based selection embedded within the inner cross-validation loop, retained feature sets contained between 4 and 9 predictors per fold (4–7 for single-source configurations, 6–9 for the fusion model), yielding a post-selection EPV of 4.1–9.3, with the upper end approaching but not meeting the conventional EPV ≥ 10 threshold and the lower end — driven by the fusion model’s larger feature set — falling well short of it. This constraint on predictor count is acknowledged as a limit on the precision of individual effect estimates.

### 3.2 Candidate biomechanical and movement-quality predictors and incremental value over injury history

Global SHAP analysis of the best-performing tree-based model — the XGBoost fusion configuration (see §3.3) — identified previous-injury history as the single largest contributor to model output (mean |SHAP| = 0.31). Among biomechanical features, the peak tibial acceleration asymmetry index (ASI-PTA) ranked second (mean |SHAP| = 0.24), with higher asymmetry values associated with elevated injury probability across nearly all observations in the dependence plot. Surrogate vertical loading rate on the non-dominant limb ranked third (mean |SHAP| = 0.19), showing a broadly monotonic positive relationship above 1.8 g·ms^−^¹. Among FMS task scores, the in-line lunge (ILL) and active straight-leg raise (ASLR) contributed most (mean |SHAP| = 0.17 and 0.14, respectively), with scores of 1 on either task associated with a consistent upward shift in predicted risk. The deep squat contributed modestly (mean |SHAP| = 0.09), and the remaining FMS tasks showed negligible SHAP mass. Sacral angular-velocity range, indexing pelvis–trunk stability, yielded a moderate positive contribution (mean |SHAP| = 0.12). These rankings, together with the direction of each predictor’s effect and its data source, are presented in **Table 1** and **Figure 1**.

**Figure 1:**
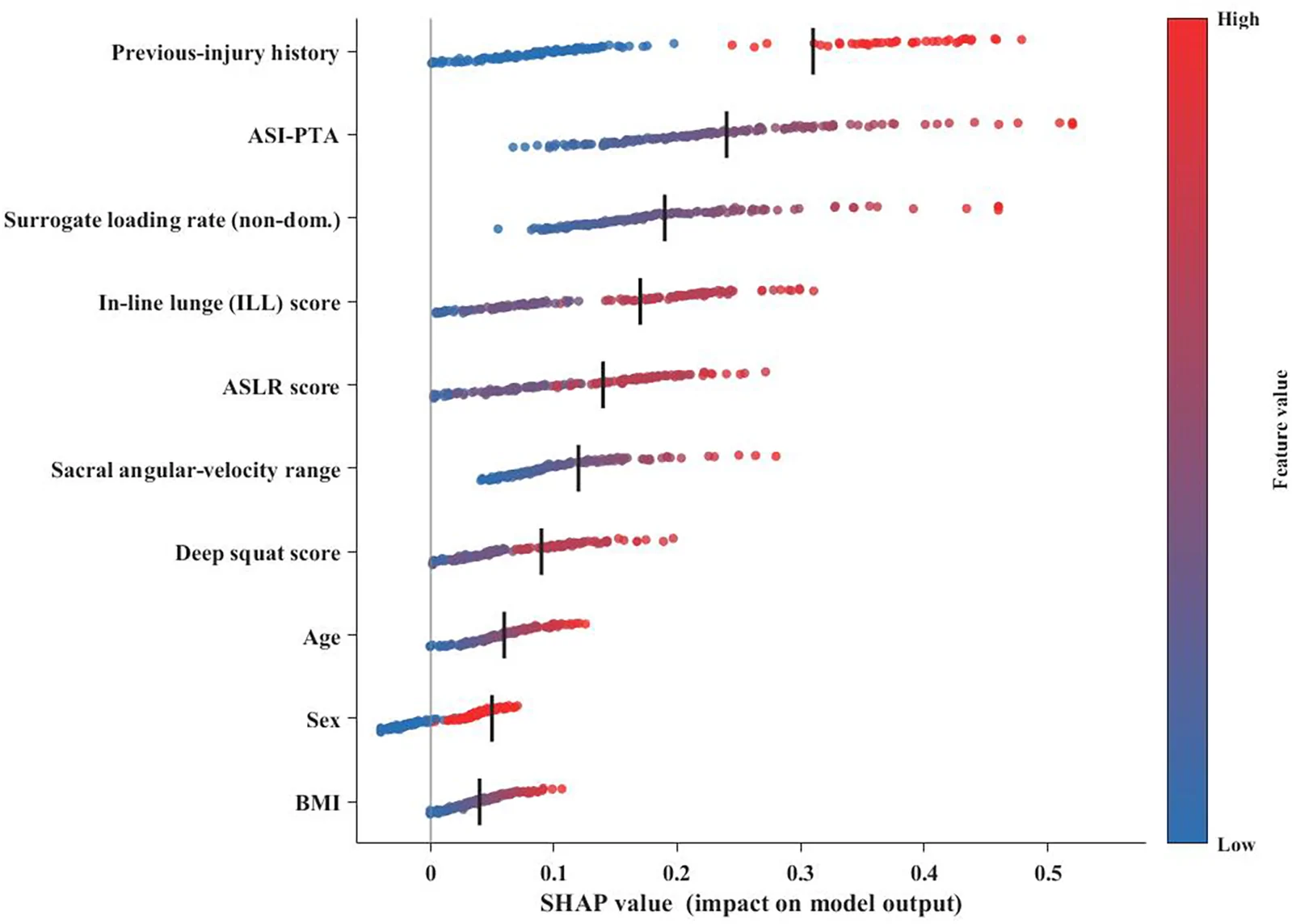
Global feature importance from the SHAP beeswarm plot of the fused XGBoost model. Each point represents one athlete; horizontal position indicates the SHAP value (positive = increased injury probability; negative = decreased injury probability); color reflects the feature value from low (blue) to high (red). Features are ordered by mean absolute SHAP value. ASI-PTA, peak tibial acceleration asymmetry index; ILL, in-line lunge; ASLR, active straight-leg raise; IMU, inertial measurement unit; FMS, Functional Movement Screen.

**Table 1** also reports the selection frequency of each feature across the 25 inner-loop LASSO folds (5 outer × 5 inner). Previous-injury history was retained in 100% of folds; ASI-PTA in 92%; surrogate loading rate on the non-dominant limb in 84%; and in-line lunge score in 80% — establishing these four features as robust predictors whose presence in the model was substantially stable across fold partitions. Active straight-leg raise was retained in 72% of folds, and sacral angular-velocity range in 64%; both contributed meaningfully to the SHAP rankings but showed moderate partition sensitivity. Deep squat (48%), age (40%), sex (32%), and BMI (24%) were retained in fewer than half of folds; their SHAP contributions should accordingly be regarded as suggestive rather than stable, consistent with their placement in the lower portion of the importance ranking. The gradient from high to low selection frequency closely mirrored the SHAP magnitude ordering, indicating that the predictor hierarchy was not driven by idiosyncratic fold configurations. As a model-independence check, regularized logistic regression yielded odds ratios of 2.39 (95% CI: 1.14–4.98) for previous-injury history, 1.84 (1.02–3.32) for ASI-PTA, 1.62 (0.91–2.88) for surrogate loading rate, and 1.55 (0.78–3.08) for in-line lunge score, preserving both the direction and relative ranking of the SHAP-derived ordering (**Supplementary Table 1**).

To assess the incremental value of biomechanical features beyond injury history, the fusion model was also estimated excluding previous injury as a covariate. The biomechanics-only configuration yielded an out-of-fold AUC of 0.63 (95% CI: 0.53–0.73), compared with 0.70 (95% CI: 0.61–0.79) when injury history was included. The AUC increment associated with adding injury history to the biomechanical feature set was 0.07 (DeLong test: *p* = 0.038), indicating that both sources carry independent predictive signal rather than redundant information.

### 3.3 Comparative performance across data-source configurations

Across the three ablation configurations, fusion of FMS-derived and wearable-derived features consistently produced the highest discriminative performance, as summarized in **Table 3** and **Figure 2**. In the out-of-fold evaluation, the XGBoost fusion model achieved an AUC of 0.70 (95% CI: 0.61–0.79) and a PR-AUC of 0.43 (95% CI: 0.31–0.55), compared with an AUC of 0.62 (95% CI: 0.52–0.72) and PR-AUC of 0.36 (95% CI: 0.25–0.48) for the FMS-only configuration, and an AUC of 0.64 (95% CI: 0.54–0.74) and PR-AUC of 0.38 (95% CI: 0.27–0.50) for the wearable-only configuration. The no-skill PR-AUC baseline for this sample (equal to the marginal injury rate) was 0.23. The AUC difference between fusion and FMS-only was 0.08 (DeLong: *p* = 0.031); between fusion and wearable-only, 0.06 (*p* = 0.047); between the two single-source configurations, 0.02 (*p* = 0.41), indicating that neither source alone dominated the other.

**Table 3:** Out-of-fold discriminative performance of four classifiers across three data-source configurations.

Configuration	Classifier	AUC (95% CI)	PR-AUC (95% CI)	Sens.	Spec.	F_1_	Events per fold
FMS-only							
	XGBoost	0.63 (0.53–0.73)	0.37 (0.26–0.49)	0.54	0.74	0.49	6–9
	Random forest	0.61 (0.51–0.71)	0.35 (0.24–0.47)	0.51	0.72	0.47	6–9
	Log. regression	0.62 (0.52–0.72)	0.36 (0.25–0.48)	0.54	0.73	0.48	6–9
	SVM	0.59 (0.49–0.69)	0.33 (0.22–0.45)	0.49	0.71	0.45	6–9
Wearable-only							
	XGBoost	0.65 (0.55–0.75)	0.39 (0.27–0.51)	0.57	0.75	0.50	6–9
	Random forest	0.63 (0.53–0.73)	0.37 (0.26–0.49)	0.54	0.74	0.48	6–9
	Log. regression	0.64 (0.54–0.74)	0.38 (0.27–0.50)	0.55	0.74	0.49	6–9
	SVM	0.61 (0.51–0.71)	0.35 (0.24–0.47)	0.51	0.72	0.46	6–9
Fusion							
	XGBoost	0.70 (0.61–0.79)	0.43 (0.31–0.55)	0.57	0.76	0.51	6–9
	Random forest	0.68 (0.58–0.77)	0.41 (0.29–0.53)	0.54	0.75	0.49	6–9
	Log. regression	0.65 (0.55–0.75)	0.39 (0.27–0.51)	0.54	0.74	0.48	6–9
	SVM	0.64 (0.54–0.74)	0.38 (0.27–0.50)	0.52	0.73	0.47	6–9

AUC, area under the receiver operating characteristic curve; PR-AUC, area under the precision–recall curve; Sens., sensitivity; Spec., specificity; F_1_, harmonic mean of precision and recall. All metrics derived from out-of-fold predictions aggregated across nested five-fold cross-validation. Bold row (Fusion/XGBoost) represents the primary model used for SHAP interpretation.

**Figure 2:**
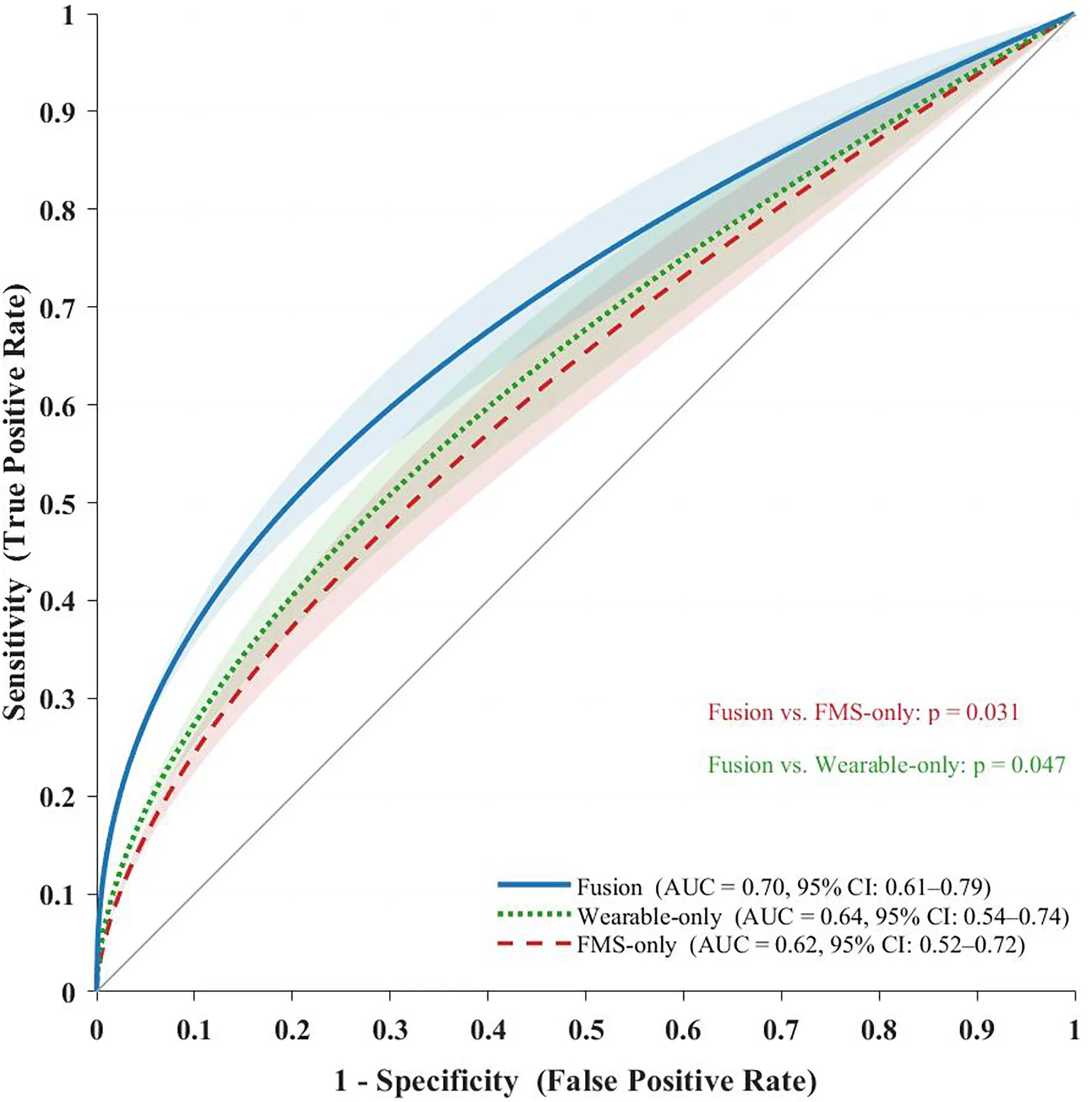
Receiver operating characteristic curves for the three data-source configurations under out-of-fold evaluation. Shaded regions indicate 95% confidence intervals estimated from the out-of-fold prediction distribution. Pairwise AUC differences were tested with the DeLong method; p-values are shown for fusion vs. FMS-only (p = 0.031) and fusion vs. wearable-only (p = 0.047). The diagonal dashed line represents the no-skill classifier. AUC, area under the receiver operating characteristic curve.

Among classifiers, XGBoost produced the highest fusion AUC (0.70), followed by random forest (0.68; 95% CI: 0.58–0.77), regularized logistic regression (0.65; 95% CI: 0.55–0.75), and SVM (0.64; 95% CI: 0.54–0.74). Confidence intervals overlapped substantially across models, and no ensemble classifier exceeded regularized logistic regression by a margin that cleared the confidence interval overlap, consistent with prior reports in prospective injury prediction literature (Jauhiainen et al., 2021). Mean retained feature counts were 5.1 (FMS-only), 5.8 (wearable-only), and 7.4 (fusion), confirming that the fusion increment arose alongside a modest rather than dramatic increase in dimensionality. Per-fold positive-event counts ranged from 6 to 9 across outer folds, and fold-level AUCs for the best-performing model spanned 0.58 to 0.79, reflecting the variability expected under a limited event count.

Sex was examined as both a pooled feature and through stratified analyses. In the male subgroup (n = 90; 19 events), fusion AUC was 0.67 (95% CI: 0.54–0.80); in the female subgroup (n = 71; 18 events), it was 0.69 (95% CI: 0.55–0.82). The confidence intervals overlapped substantially and neither stratum yielded a stable performance advantage, so stratified models were not pursued further. This is consistent with the available sample size being insufficient to support reliable sex-specific modeling, a limitation addressed in §4.6.

### 3.4 Model reliability and calibration

Calibration of the fusion XGBoost model, evaluated on the original non-resampled class distribution (23.0% positives), yielded a Brier score of 0.171. For reference, a no-skill model assigning the marginal base rate to all observations would produce a Brier score of 0.177 in this sample, calculated as the product of the marginal injury rate and its complement, placing the model’s improvement above the uninformative baseline, though modest in absolute magnitude. At the threshold maximizing the sum of sensitivity and specificity in the out-of-fold predictions, the model achieved sensitivity of 0.57 (95% CI: 0.40–0.73), specificity of 0.76 (95% CI: 0.68–0.84), positive predictive value of 0.46, and an F_1_ score of 0.51. A SMOTE sensitivity analysis yielded a marginal AUC improvement of +0.02 relative to the unweighted model, while Brier scores on the original distribution were 0.171 (with SMOTE) and 0.178 (without), indicating that the resampling approach did not materially distort calibration when evaluated on the correct distribution. The confusion matrix and calibration summary for the fusion model are presented in **Figure 3**.

**Figure 3:**
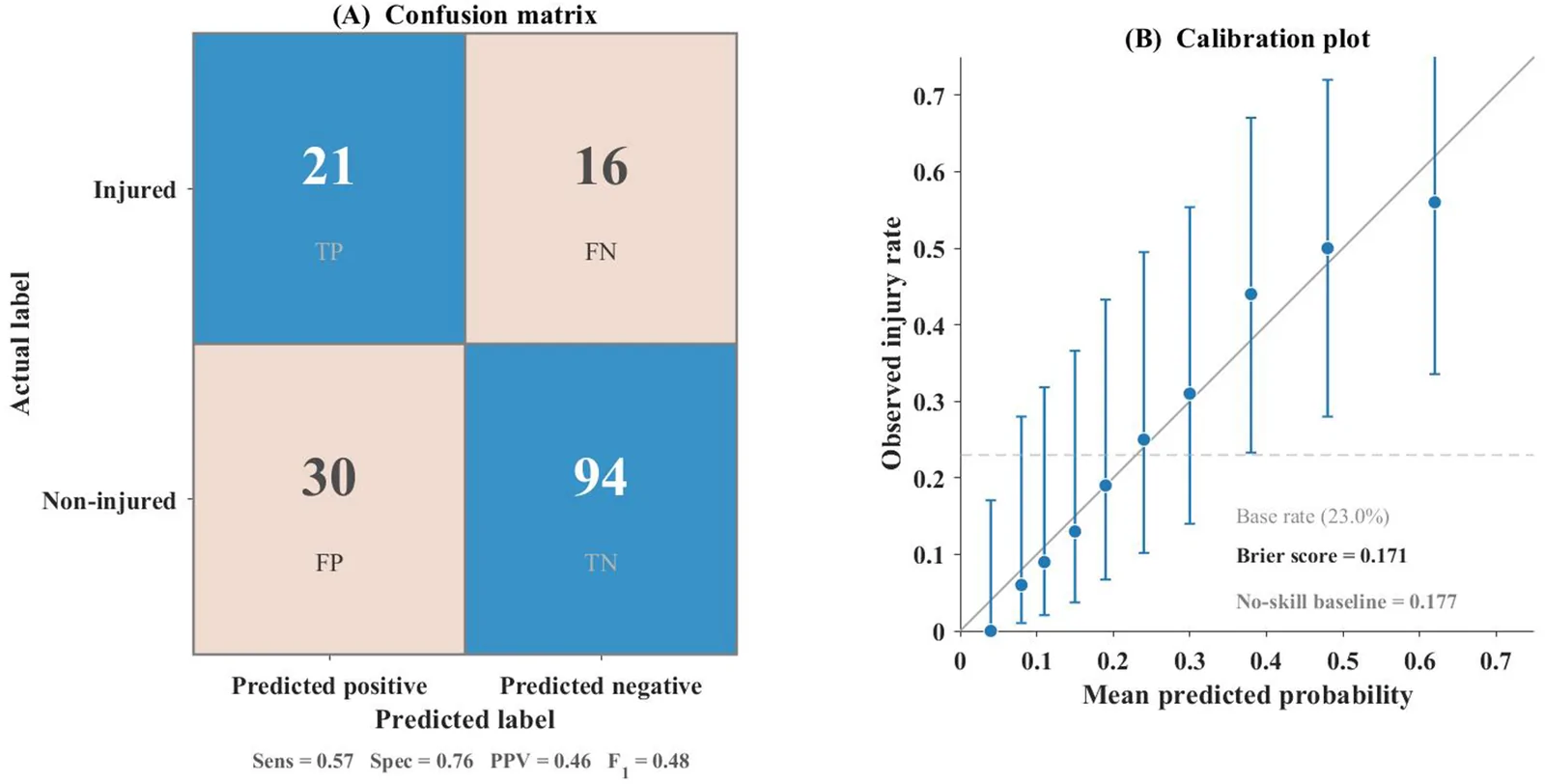
Model reliability and calibration of the fused XGBoost model evaluated on the original, non-resampled class distribution (23.0% positives). **(A)** Confusion matrix at the threshold maximizing the sum of sensitivity and specificity; correct predictions (TP, TN) are shown in blue and incorrect predictions (FP, FN) in beige. **(B)** Calibration plot of mean predicted probability against observed injury rate across deciles; error bars represent 95% Wilson confidence intervals; the diagonal line represents perfect calibration; the dashed horizontal line indicates the marginal base rate (23.0%). Brier score = 0.171; no-skill baseline = 0.177. TP, true positive; TN, true negative; FP, false positive; FN, false negative; Sens., sensitivity; Spec., specificity; PPV, positive predictive value.

A chronological split — training on the first 18 weeks of the season and testing on the remaining 12 weeks — was examined as a supplementary sensitivity check. The later window contained 11 injury events, which was insufficient to support stable fold-level AUC estimation; results are therefore not reported numerically but the analysis confirmed that no systematic performance reversal was observed between early- and late-season prediction windows.

### 3.5 Exploratory cross-source interaction effects

The SHAP interaction analysis identified one moderately stable cross-source interaction: elevated ASI-PTA co-occurring with an ILL score of 1 was associated with an additive upward shift in predicted risk beyond the main effect of either feature alone (mean interaction SHAP = 0.09). Across 200 permutation-resampled subsets — a number selected to balance computational tractability with stability estimation reliability at this sample size — this interaction was retained in 71% of iterations, meeting the pre-specified 70% directional stability threshold. A secondary interaction between surrogate loading rate and ASLR score was directionally consistent but fell marginally below the stability criterion (68% retention) and is therefore presented in the **Supplementary Figure 1** without interpretation. No other cross-source interaction exceeded the stability threshold. The ASI-PTA × ILL interaction dependence plot is shown in **Figure 4**.

**Figure 4:**
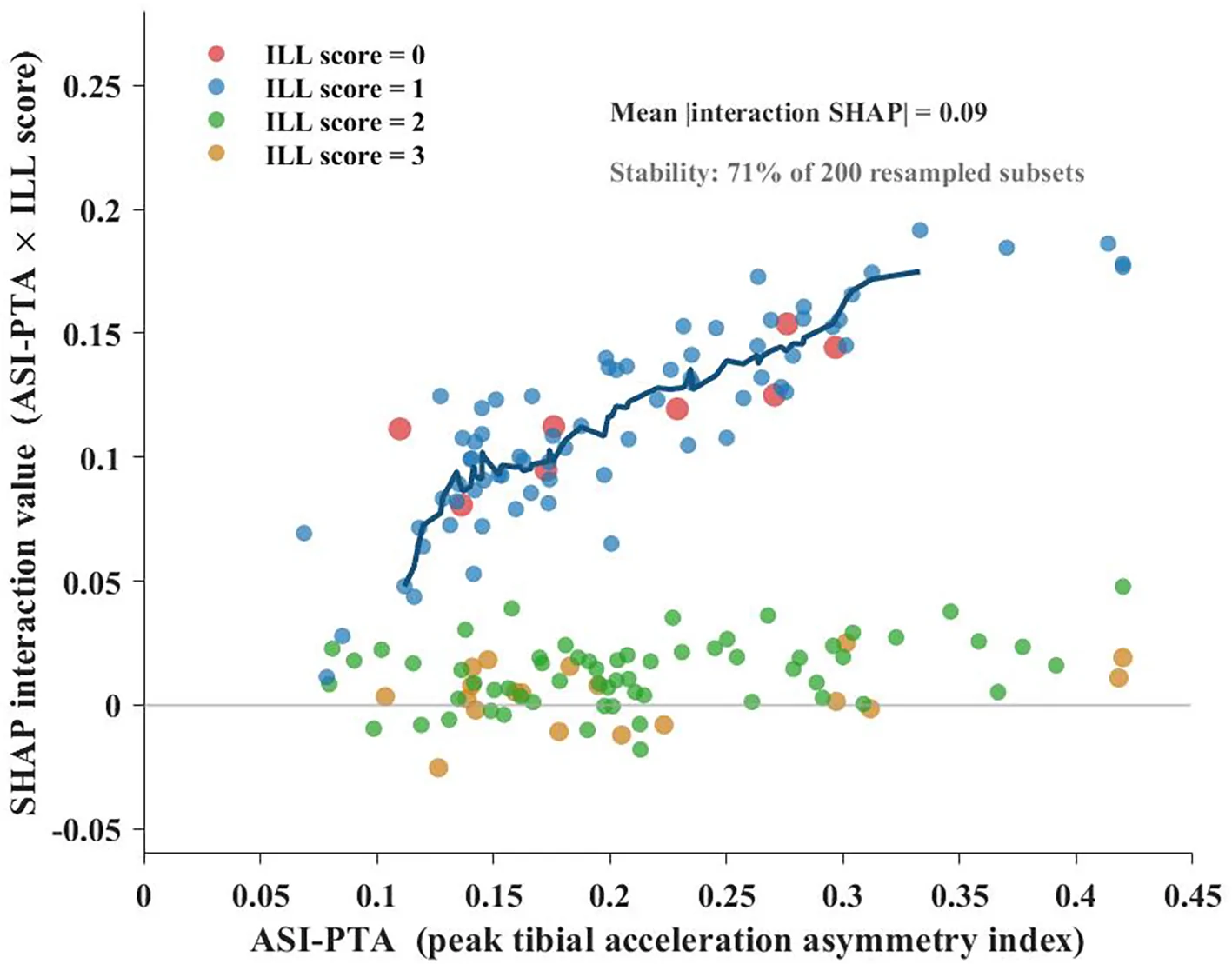
Exploratory SHAP interaction dependence plot for ASI-PTA and in-line lunge (ILL) score. Each point represents one athlete; the x-axis shows the ASI-PTA value; the y-axis shows the SHAP interaction value between ASI-PTA and ILL score. Point color indicates ILL task score (0–3). The solid trend line represents a moving-average smoother for ILL score = 1, the group with the strongest interaction effect. This interaction was retained in 71% of 200 permutation-resampled subsets, meeting the pre-specified 70% directional stability threshold. ASI-PTA, peak tibial acceleration asymmetry index; ILL, in-line lunge; SHAP, SHapley Additive exPlanations.

## 4 Discussion

### 4.1 Principal findings

Three interconnected findings emerge from this study. Among the biomechanical and movement-quality features examined, peak tibial acceleration asymmetry and surrogate vertical loading rate on the non-dominant limb carried the strongest predictive signal, with the in-line lunge and active straight-leg raise contributing meaningfully among the FMS task scores — a pattern that points toward inter-limb loading imbalance and neuromuscular control deficit as the operative mechanisms rather than global movement quality or absolute impact magnitude alone. Previous-injury history remained the dominant single predictor, yet biomechanical features contributed an independent AUC increment of 0.07 when added to injury history as a covariate, suggesting that what the sensors and movement screen capture is not simply a proxy for prior damage but reflects something physiologically distinct about an athlete’s current load-tolerance profile. The fusion of wearable-derived and FMS-derived features outperformed either source in isolation — modestly but consistently and with statistical support from pairwise DeLong comparisons — and the retained feature counts across configurations confirmed that this advantage reflected informational complementarity rather than dimensionality alone. Calibration was above the no-skill baseline but modest, and the exploratory interaction between asymmetry index and in-line lunge score met the pre-specified directional stability threshold, whereas the loading-rate–ASLR interaction did not — an outcome reported as such rather than reinterpreted. Taken together, these results are best read not as a validated clinical tool but as a structured empirical window into which quantifiable biomechanical signals carry load-related injury risk information in collegiate athletes, and under what conditions combining them adds value. The overlapping confidence intervals between XGBoost and logistic regression preclude a claim of statistical superiority for the ensemble classifier; the XGBoost model was retained for SHAP analysis because interaction decomposition requires a non-additive architecture, not because of a discrimination advantage. The logistic regression odds ratios (**Supplementary Table 1**) confirm that this ranking is not model-dependent.

### 4.2 Physiological interpretation of key predictors

The primacy of inter-limb asymmetry in tibial impact loading — rather than absolute peak acceleration on either limb — warrants careful physiological unpacking. Bone adapts to habitual mechanical stimulation through a remodeling cycle in which osteoclastic resorption precedes osteoblastic formation, and it is the temporal mismatch between these two phases that creates a window of elevated fracture susceptibility when loading accumulates faster than repair can follow (Hoenig et al., 2022). What asymmetric tibial loading implies is not simply that one limb receives more force, but that the spatial and directional distribution of strain across the tibial cortex diverges from the pattern to which the bone has adapted over prior training. A limb that habitually absorbs less impact is structurally tuned to a lower loading regime; when competitive demands redistribute that burden — through fatigue-induced gait asymmetry, compensatory movement strategies compensatory movement strategies, or sport-specific positional demands — the under-prepared limb encounters a strain environment that its current remodeling state cannot buffer (Warden et al., 2021). The monotonically increasing SHAP dependence observed above approximately 1.8 g·ms^−^¹ for surrogate loading rate on the non-dominant limb is consistent with this interpretation: it is not loading per se but loading that exceeds the tissue’s adaptive history that places it at risk.

Tendon responds to mechanical stimulation through a comparable but slower adaptive process. Collagen synthesis and cross-link maturation following a loading stimulus require days to weeks, meaning that the structural capacity of patellar and Achilles tendons lags measurably behind acute changes in mechanical demand (Kjaer et al., 2009). When the loading rate increases abruptly — as it typically does during intensified competition phases — the tendon is asked to transmit forces that its current collagen architecture has not yet reorganized to handle efficiently (Magnusson et al., 2008). The SHAP contribution of surrogate loading rate is therefore physiologically coherent whether the outcome is a tendinopathy or a tibial stress injury, since both reflect exposure outpacing adaptation, differing only in the tissue through which that mismatch is expressed.

The FMS task scores that contributed most — the in-line lunge and the active straight-leg raise — are not arbitrary selections from a seven-item battery. The in-line lunge loads a split-stance position that demands coordinated hip and ankle mobility alongside frontal-plane stability, and a score of 1 on this task flags an inability to perform this under bodyweight alone; under the far greater demands of running and cutting, such a deficit is plausibly expressed as altered ground contact mechanics and compensatory loading redistribution. The active straight-leg raise captures hamstring and posterior chain extensibility in a position that isolates the contralateral limb from compensatory pelvic tilt — a constraint that makes it sensitive to the kind of asymmetric flexibility limitation that may not be apparent in bilateral assessments. That these two tasks, rather than the composite FMS score, carried predictive weight aligns with the argument that collapsing seven movement patterns into a single total compresses precisely the pattern-specific information that carries physiological signal (Eckart et al., 2025). The interaction between ASI-PTA and ILL score — the one cross-source combination that met the directional stability criterion — is mechanistically plausible on these grounds: an athlete who both loads asymmetrically during locomotion and demonstrates unilateral movement-quality compromise under a quasi-static challenge is likely operating closer to the load-tolerance boundary on the affected side than either measure alone would suggest.

Tibial accelerometry provides a peripheral surrogate for impact loading. While higher surface-measured accelerations correlate with greater ground reaction force magnitudes, the relationship between tibial surface acceleration and stresses distributed within cortical bone is indirect, affected by soft-tissue damping, gait pattern, and bone geometry that three sensors cannot resolve (Sheerin et al., 2019). The SHAP values reported here reflect predictive contributions of these surrogate signals within this model and this sample; they do not constitute direct evidence that bone stress exceeded a given threshold in injured athletes (Xiang et al., 2024). The data are consistent with an asymmetric load-adaptation mismatch mechanism, but confirming it would require internal bone strain measurement or validated finite-element modeling beyond the scope of wearable-based field assessment.

### 4.3 Incremental value of biomechanical fusion over injury history

Previous injury ranked first in global SHAP importance and its inclusion added 0.07 AUC units beyond the biomechanics-only model. The more substantive question is what the biomechanical features contribute over and above that history. Injury history functions primarily as a summary of accumulated tissue vulnerability: a prior stress fracture or tendinopathy leaves behind remodeled bone, reorganized collagen architecture, and potentially altered motor patterns that persist well into subsequent seasons. What it cannot capture is the athlete’s current mechanical exposure profile — how asymmetrically load is being distributed across limbs at the time of assessment, how the neuromuscular system is organizing movement under the specific demands of the upcoming competitive season, or whether the gap between habitual loading and tissue adaptive capacity has widened since the last injury event. Biomechanical features derived from IMU monitoring and movement screening occupy precisely this space: they index the present state of the load–capacity relationship rather than its history. That the two sources contribute independently to the model, confirmed by the DeLong comparison (DeLong et al., 1988), is therefore not a statistical curiosity but a reflection of the fact that they measure genuinely different aspects of injury risk — one retrospective and structural, the other prospective and functional.

The fusion increment of 0.08 AUC units over FMS-only and 0.06 over wearable-only, while statistically significant, sits at the modest end of what the literature reports for multi-source fusion approaches in comparable settings. This should be interpreted carefully. A fusion advantage that is real but small may mean that the two sources share more variance than their conceptual distinctness implies, or that the event count in this sample constrained the model’s ability to fully exploit cross-source complementarity — a possibility that cannot be excluded given the post-selection EPV of 4.1–9.3. It does not, however, imply that the fusion approach is without value: a consistent, reproducible increment across all pairwise comparisons, supported by stable feature counts that rule out a dimensionality artefact, provides reasonable grounds for treating the two data streams as genuinely complementary rather than redundant.

Where the fusion increment was negligible or absent — in the SVM configuration and in the sex-stratified subgroups — the most parsimonious explanation is statistical rather than biological: too few events per stratum to allow the model to learn the cross-source relationships that drive the advantage in the pooled analysis. This suggests that the fusion benefit is contingent on adequate data to exploit it and should not be assumed to generalize automatically to smaller monitoring programs without re-evaluation.

### 4.4 Comparison with existing literature

The discriminative performance observed here — fusion AUC of 0.70 under nested cross-validation — sits within the range reported by prospective studies that have applied machine learning to lower-limb injury prediction using wearable or multimodal data, though toward the conservative end of that range, This positioning is expected: nested cross-validation with embedded feature selection is deliberately conservative, and the AUC values it produces are more likely to reflect true out-of-sample performance than estimates derived from single train-test splits or non-nested procedures. The field has been slow to adopt this discipline, and performance figures that appear impressive in isolation frequently shrink considerably once evaluated under conditions that prevent information leakage (Van Eetvelde et al., 2021).

The finding that XGBoost outperformed regularized logistic regression by only a narrow, confidence-interval-overlapping margin is consistent with results from prospective cohort work in young team-sport athletes, where the discriminative advantage of ensemble methods over simpler linear classifiers proved similarly modest, with AUC values for both approaches converging around 0.63 (Jauhiainen et al., 2021). That study also highlighted the difficulty of achieving stable injury prediction in populations where event counts constrain model complexity, a constraint that operated here as well. Where the present study diverges most clearly from prior work is in its treatment of the FMS. Most previous applications have relied on the composite score as the sole movement-quality input, a choice whose limitations are well documented (Eckart et al., 2025), whereas retaining individual task scores here allowed the model to identify which specific movement patterns — rather than the aggregate — carried predictive weight. The explainability framework developed around trunk-mounted accelerometry in a sex-stratified design reported AUCs of 0.615 and 0.645 for female and male subgroups respectively (Bogaert et al., 2022), values that align closely with the single-source wearable AUC of 0.64 observed here and lend external plausibility to that estimate. The sex-stratified performance gap reported in that work — where female-specific models outperformed pooled models — was not reproduced in the present data, most likely because the per-stratum event counts were insufficient to realize whatever advantage stratification might offer; the possibility that sex modifies the predictive structure of these features therefore remains open rather than resolved.

The fusion advantage documented here — 0.08 AUC units over FMS-only, with a statistically significant DeLong comparison — echoes the precision and recall improvements reported for multimodal fusion over single-modality approaches in a lower-extremity non-contact injury prediction study using interpretable machine learning, where combining movement and loading features yielded consistent gains over either stream alone (Huang et al., 2022). That study’s design is the closest structural parallel to the present work among published literature, and the convergence of findings across two independently collected datasets strengthens confidence that the fusion increment reflects a genuine signal rather than a sample-specific artefact. One meaningful difference is that the earlier work did not embed feature selection within cross-validation, raising the possibility that some of its reported advantage reflects selection optimism; the present design addresses this explicitly.

The use of GPS-derived workload data combined with decision-tree modeling in professional soccer demonstrated that prospective wearable-based prediction is feasible at the team level, achieving recall of 0.80 at a precision of 0.50 (Rossi et al., 2018) — a precision figure lower than the 0.46 PPV observed here despite a substantially larger and more homogeneous sample. This comparison underscores that precision in injury prediction remains difficult regardless of modality or population. Explainable machine learning applied to biomechanical features in professional soccer similarly found that movement asymmetry and loading-related variables dominated SHAP importance rankings (Calderón-Díaz et al., 2024), a convergence that lends cross-sport support to the asymmetry-centered interpretation developed in §4.2, even though the populations, sports, and sensor configurations differed considerably between studies.

### 4.5 Clinical and translational implications

The performance metrics reported here — a fusion AUC of 0.70, sensitivity of 0.57, and positive predictive value of 0.46 — do not support the use of this framework as a standalone clinical screening tool, and claiming otherwise would misrepresent what the data can bear. At the threshold that maximized the sensitivity-specificity balance, more than half of flagged athletes did not sustain an injury, and more than four in ten injured athletes were not identified in advance. These figures are characteristic of injury prediction models in real-world prospective settings, where base rates are moderate and predictors are imperfect surrogates for underlying physiology (Bittencourt et al., 2016). The appropriate frame for interpreting these results is therefore not clinical diagnosis but biomechanical phenotyping — the identification of movement and loading patterns that, at the population level, are associated with elevated risk and that carry a physiological rationale worth acting on in monitoring practice.

Within that frame, the findings carry practical implications for how preseason assessment data might be used. An athlete presenting with marked inter-limb asymmetry in tibial impact loading alongside a score of 1 on the in-line lunge represents a specific, actionable profile: the combination points toward unilateral loading redistribution under dynamic demand and a movement-quality deficit under quasi-static challenge that together suggest a widened gap between mechanical exposure and neuromuscular control capacity. Whether that gap is addressed through load management — moderating the acute-to-chronic workload ratio during the early competitive phase (Gabbett, 2016) — or through targeted movement intervention depends on clinical judgement that the model cannot replace, but the model can make the relevant profile visible in a way that neither source alone achieves. The surrogate loading rate on the non-dominant limb adds a further layer: where that signal is elevated and asymmetric, the tissue adaptation logic outlined in §4.2 suggests that the non-dominant limb may be absorbing a loading history it has not been conditioned to handle, a situation that modified training distribution or technique cuing might partially address (Maupin et al., 2020).

In practical terms, three integration steps follow. First, retain task-level FMS scores — particularly in-line lunge and active straight-leg raise — rather than relying on the composite total alone. Second, for athletes scoring 1 on either task, add a brief standardized running trial with the three-sensor IMU configuration to quantify inter-limb impact asymmetry. Third, when both indicators are abnormal, consider prioritizing the athlete for closer early-season volume monitoring rather than automatic restriction. This staged approach treats the model as an input to clinical reasoning, not an automated rule, and rests on population-level associations rather than individually validated thresholds.

Two cautions temper these translational statements. The cohort was drawn from a single institution and followed for one competitive season, so the biomechanical profiles identified here as risk-associated reflect the load demands and movement culture of that specific athletic environment. Whether the same features carry equivalent weight in distance runners, swimmers, or contact-sport athletes operating under different loading regimes is an empirical question that the present data cannot answer. Equally, the SHAP values reported here describe average marginal contributions across the sample; they do not constitute validated individual-level risk scores, and applying them as such — without the out-of-fold validation infrastructure that produced them — would represent an extrapolation beyond what the analysis supports. Elite athletes with higher training volumes, youth athletes with skeletal immaturity, and recreational populations with different movement demands each represent contexts in which the predictor weights identified here cannot be assumed to hold.

### 4.6 Limitations and future directions

Several limitations constrain the conclusions that can be drawn from this work, and acknowledging them precisely is more useful than cataloguing them generically. The most consequential is the event count. With 37 outcome events across 161 athletes, the post-selection EPV remained below 10 in most folds, which limits the precision of individual predictor effect estimates and reduces the power of the DeLong comparisons to detect modest AUC differences. The fusion increment and the SHAP rankings reported here are directionally plausible and internally consistent, but they should be treated as hypothesis-generating rather than definitive — the confidence intervals are wide enough that a replication study with a larger event pool could reasonably shift the relative standing of individual predictors without contradicting the broader pattern. The scoping review landscape for machine learning in sport injury prediction makes this point at the field level, noting that small samples and limited event counts remain the dominant methodological constraint across the literature (Leckey et al., 2025), and the present study is not exempt from that critique. The fold-level AUC range of 0.58–0.79 reflects this instability directly, and residual optimism cannot be excluded at this sample size; the fusion AUC of 0.70 is best understood as a central-tendency estimate rather than a precise benchmark.

The outcome definition, while deliberate, introduces its own limitations. Grouping tibial stress injuries, tendinopathies, and muscle strains into a composite load-related endpoint was justified on the grounds that all share a common load–capacity mechanism, but the heterogeneity within that grouping means that a single predictive model is being asked to represent injury processes that differ in their tissue substrate, time course, and sensitivity to specific biomechanical features. Whether asymmetry in tibial impact loading predicts stress fractures through a different pathway than it predicts Achilles tendinopathy — and whether that difference matters for the SHAP rankings — cannot be determined from the present data. Disaggregating the outcome by injury subtype would require a substantially larger cohort than was available here. Tibial impact asymmetry has the most direct physiological link to bone stress through asymmetric strain accumulation; its connection to tendinopathy likely operates through loading-rate effects on collagen turnover, whereas its relevance to muscle strain may be mediated by compensatory movement strategies rather than tibial mechanics per se. With 8–11 events per subtype, these pathway differences could not be tested, and this aggregation should be weighed when interpreting the predictor rankings.

A separate limitation concerns the absence of in-season workload data. Training volume, intensity, and acute-to-chronic load ratios were not monitored during follow-up, so it cannot be determined whether the identified biomechanical features reflect intrinsic risk or are partly confounded by differential training exposure. This omission was deliberate: the study tested whether a preseason biomechanical snapshot carries prospective information, a question distinct from modelling dynamic load–capacity interactions. It does, however, limit the SHAP values to statistical associations rather than causal pathways. Future work should integrate continuous load monitoring to evaluate whether preseason predictors are attenuated once cumulative exposure is accounted for.

The absence of an external validation cohort is a further constraint that the out-of-fold procedure, however carefully implemented, cannot fully substitute for. Internal validation establishes that the model generalizes beyond the training data within this sample; it does not establish that the identified predictors or their relative weights would replicate in athletes from a different institution, competitive level, or sport. External validation in an independent sample — ideally drawn from a different athletic environment — remains the necessary next step before any translational claim can be made with confidence. Until such replication is completed, the reported metrics, predictor rankings, and interaction effects should not be assumed to generalize across populations, competitive levels, or institutional settings.

Several design features that were either infeasible or underpowered in the present study point toward productive directions for future work. Sex-specific modeling was examined but could not be pursued reliably given per-stratum event counts of 18–19; a larger prospective cohort would allow the question of whether biomechanical risk profiles differ between male and female athletes to be answered rather than deferred. Extending the monitoring window beyond a single competitive season would address both the event count limitation and whether preseason biomechanical profiles retain predictive relevance across successive years of training adaptation. The exploratory interaction between asymmetry index and in-line lunge score met the directional stability criterion here, but its clinical and physiological interpretation would benefit from replication in an independent sample before it is treated as more than a hypothesis worth testing. More broadly, the field would benefit from prospective studies that pair wearable-based surrogates with direct tissue-level assessment — such as bone scintigraphy, tendon ultrasonography, or musculoskeletal modeling — to close the inferential gap between sensor-derived proxies and the mechanical environment experienced by the tissue (Xiang et al., 2024; Leckey et al., 2025). Multicenter designs, repeated within-season biomechanical assessments, integration of psychological and sleep-recovery variables, and deep-learning models applied to continuous sensor streams represent further avenues that could strengthen the translational value of this prediction framework.

## 5 Conclusion

This study set out to identify quantifiable biomechanical and movement-quality features that carry the signal of lower-limb load-related injury in collegiate athletes, and to examine whether combining wearable-derived and FMS-derived information adds discriminative value beyond what either source provides alone. The results support an affirmative answer to both questions, with important qualifications attached to each.

Inter-limb asymmetry in tibial impact loading and surrogate vertical loading rate on the non-dominant limb emerged as the biomechanical features with the strongest and most consistent predictive contribution, with the in-line lunge and active straight-leg raise scores adding meaningful signal among the FMS task inputs. That these features — rather than absolute loading magnitude or global movement quality — dominated the SHAP rankings is physiologically coherent: it is the mismatch between the loading a tissue has been conditioned to absorb and the loading it is actually asked to bear, not the loading level in isolation, that drives the adaptive failure underlying stress injuries and tendinopathies. Previous-injury history remained the single largest predictor, as expected, yet the biomechanical feature set contributed an independent increment beyond it, suggesting that wearable sensing and movement screening together capture aspects of the current load–capacity relationship that injury history alone cannot reflect.

The fusion of both data sources outperformed either in isolation consistently and with statistical support, and the retained feature counts across configurations confirmed that this advantage was not an artefact of dimensionality. The magnitude of the increment was modest — as it should be in a setting where event counts limit what any model can learn — and no ensemble classifier exceeded regularized logistic regression by a margin that cleared the confidence interval overlap, consistent with prior reports in prospective injury prediction literature (Jauhiainen et al., 2021), a finding that cautions against overinvesting in algorithmic complexity when the fundamental constraint is data volume rather than model expressivity. The one cross-source interaction that met the pre-specified directional stability criterion — elevated tibial impact asymmetry co-occurring with a compromised in-line lunge score — is mechanistically plausible and worth carrying forward as a testable hypothesis, while the secondary loading-rate–ASLR interaction did not meet that threshold and is not interpreted here.

The overall discriminative performance, a fusion AUC of 0.70 under nested cross-validation, falls within the realistic ceiling that prospective injury prediction studies have repeatedly encountered, and the calibration improvement over the no-skill baseline, while present, was modest. These figures do not support clinical deployment as a screening instrument but do support the use of this framework as a structured approach to biomechanical phenotyping — making visible, at the individual athlete level, the specific combination of loading asymmetry and movement-quality deficit that population-level analysis associates with elevated risk. Whether that visibility translates into reduced injury burden depends on what practitioners do with it, a question that lies beyond prediction modeling and belongs to intervention research. What the present work establishes is that the relevant features are quantifiable with accessible technology, that combining them adds information in a physiologically interpretable way, and that the computational framework used here — with its explicit anti-leakage design, calibration evaluation, and pre-specified stability criteria for exploratory findings — provides a methodological template that future larger-scale prospective studies can build on. Independent external replication remains a prerequisite before these findings can inform practice.

## Data Availability

The datasets presented in this article are not readily available because of institutional data governance requirements. Requests to access the datasets should be directed to Xuqingfeng Xu, hnbd5274@outlook.com.

## Supplementary material

Supplementary Table 1 Regularized logistic regression coefficients and odds ratios for features retained in ≥ 80% of inner-loop LASSO folds. β, penalized logistic regression coefficient; OR, odds ratio (exponentiated β); 95% CI, 95% confidence interval for the odds ratio derived from out-of-fold prediction variance. Direction indicates whether higher feature values (or, for ordinal FMS scores, a score of 1 vs. ≥ 2) were associated with increased injury probability — consistent with the SHAP-derived direction reported in **Table 1**. SHAP rank refers to the position in the mean |SHAP| ordering from the fused XGBoost model. Only features retained in ≥ 80% of inner-loop LASSO folds (n = 25) are included; features below this threshold were excluded to maintain consistency with the ≥ 80% retention criterion described in §2.5. ASI-PTA, peak tibial acceleration asymmetry index; ILL, in-line lunge; IMU, inertial measurement unit; FMS, Functional Movement Screen.
