# Supplementary material for: Biomechanical and movement-quality predictors of lower-limb load-related injury in collegiate athletes: a prospective cohort study using functional movement screening, wearable inertial sensing, and explainable machine learning
Source: Front Physiol. 2026 Sep 30;17:1920109. doi: 10.3389/fphys.2026.1920109 (PMC13661321; doi:10.3389/fphys.2026.1920109)
Supplement: Supplementary file 1 [file Table1.docx]

**Supplementary Materials**

**Supplementary Table 1.** Regularised logistic regression coefficients and odds ratios for features retained in ≥ 80% of inner-loop LASSO folds.

| Feature | Source | *β* coefficient | OR | 95% CI | Direction | SHAP rank |
| --- | --- | --- | --- | --- | --- | --- |
| Previous-injury history | Covariate | 0.87 | 2.39 | 1.14–4.98 | Positive | 1 |
| ASI-PTA | IMU | 0.61 | 1.84 | 1.02–3.32 | Positive | 2 |
| Surrogate loading rate (non-dominant) | IMU | 0.48 | 1.62 | 0.91–2.88 | Positive | 3 |
| In-line lunge score (ILL) | FMS | 0.44 | 1.55 | 0.78–3.08 | Positive (score = 1) | 4 |

*β*, penalised logistic regression coefficient; OR, odds ratio (exponentiated β); 95% CI, 95% confidence interval for the odds ratio derived from out-of-fold prediction variance. Direction indicates whether higher feature values (or, for ordinal FMS scores, a score of 1 vs. ≥ 2) were associated with increased injury probability — consistent with the SHAP-derived direction reported in Table 1. SHAP rank refers to the position in the mean |SHAP| ordering from the fused XGBoost model. Only features retained in ≥ 80% of inner-loop LASSO folds (n = 25) are included; features below this threshold were excluded to maintain consistency with the ≥ 80% retention criterion described in §2.5. ASI-PTA, peak tibial acceleration asymmetry index; ILL, in-line lunge; IMU, inertial measurement unit; FMS, Functional Movement Screen.
